# Some Account of Cretinism, and the Institution for Its Cure on the Abendberg, near Interlachen, in Switzerland

**Published:** 1844-04

**Authors:** 


### Art. XVI.

*Some Account of Cretinism, and the Institution for its Cure on the Abendberg, near Interlachen, in Switzerland.* By W. Twining, m.d. —*London*, 1843. 8vo.

Felix Platner in 1611 thus graphically pictured those miserable beings who still, as then, vegetate in the valleys of Switzerland, as if Nature, delighting in strong contrasts, had given human pride a lesson of humility, by placing amidst some of the most exquisite of earthly scenes the lowest, loathsomest specimens of human nature: "There are," says Platner, "some stupid creatures who besides being born so, have other vices of conformation. They are chiefly seen in the valleys, sitting at the doors of the cottages, staring upwards, or playing with sticks in their hands, and grinning at passers-by. Their heads are mis-shapen, and mouths and tongues so thick and swollen that many are unable to articulate sounds. They were indeed hideous to see."

These are the Cretins of the present day, the numbers of whom, it is supposed, amounts to 8000 completely idiotic, and double or treble that number who are more or less affected. A statistical survey, however, has been undertaken, which will give some certain information.

The word 'Cretin' is said to be derived from 'cræetira,' which, in the Romance or old Italian language, (still prevalent in a part of the canton Graubündten,) means "a poor creature;" a word very significant of a disease which consists essentially of such an enfeebled state of both body and mind as unfits them for the commonest purposes of life. "The sole condition which prevails in all is a want of tone or energy, evident either in the whole being or in a particular series of organs." Cretinism and goïtre have often been confounded, as one and the same disease. They commonly coexist; but there are many 'Cretins' that have not 'goïtre,' and vice versâ. In extreme cases the head is mis-shapen, the limbs and body deformed; the Cretin can neither hold up his head, stand, or walk; he is deaf and, consequently, dumb; his eyes give him no definite sensation; and his taste, smell, and touch are similarly defective: he is so dependent on others "that a day of neglect would be the day of his death."

Dr. Guggenbuhl, a Swiss protestant physician, struck with the wretchedness of these beings, has, with a zeal, devotion, and earnestness of purpose worthy of all praise, devoted his life to the amelioration of their condition. For two years he lived in one of the small valleys in which Cretinism was endemic to study the disease; he then travelled over Switzerland to ascertain its localities; and having satisfied himself as to the means of cure, he called the attention of his countrymen to the subject, and has been enabled to begin his benevolent experiment on a small scale.

Dr. Guggenbuhl believes that in four cases out of five the disease consists in a want of due bodily vigour, which renders the senses incapable of conveying external impressions to the mind; and not in the non-existence of the mental faculties; or, in other words, in no organic defect of the material organ of the mind; and his treatment consists in improving the bodily health by air, exercise, diet, frictions, baths, and medicines, and in subsequently rousing the inert senses by a steady course of instruction. The only cause which he has found to be constant in all those localities in Switzerland where Cretinism is endemic, is the damp warm air of close valleys among the mountains where there is no free circulation ; and it is an essential feature of his plan, that the institutions for the cure of this disease should be situated in high mountains, far above the altitude of any of the valleys in which it prevails. For this purpose he has purchased a cottage on the Abendberg, near Interlachen, 3600 feet above the level of the sea, which is 1000 feet above any place where Cretinism is endemic. Ascending a steep mountain, amongst thick fir forests, the traveller at last arrives at an open space of grass-land, on which is a cottage—a spot of rare beauty, with ranges of mountains covered with snow above, and far beneath, a lake and the green valley of Interlachen. This cottage is the field of this Swiss physician's self-denying labours. "I myself," he wrote to his countrymen, when urging the claims of these poor idiots, "will dedicate my life and all my powers to this sadly-neglected class of mankind ; and, regardless of all difficulties, will strive to realize the wish which day and night is the continual subject of my thoughts." And here he is carrying out his heroic purpose, surrounded by children in every stage of imbecility of body and of mind. He has an assistant who has had experience in the instruction of the deaf and dumb, and "Sisters of Charity" act as nurses. The bodily health is first attended to, and the fresh mountain air, proper food, cold baths, and frictions soon enable those to walk and exert their muscles who on their entrance could not walk steadily or feed themselves, or even could not hold up their heads or move their limbs. As soon as the health is sufficiently improved, education is begun. The ear is first roused by speaking through ear-trumpets, beginning with the vowels. The child is then taught to perform with its mouth the motions required to express the sound, so as to connect the sound itself with the mode of expressing it, so that the pronunciation is by degrees attained. Letters are carved in wood, by means of which the child connects them with the sounds, either by touch or sight. Thus the children gradually form words which they utter. Next common utensils—as knives, keys, &c.—are painted, and they learn to place the instruments themselves on the pictures. Sometimes, when this process does not avail to fix the sight on an object, marks or letters are figured with phosphorus on the walls of a room, and then the instruction begins, in winter, after sunset, or in summer, in a darkened room. And this method often proves effectual, when others fail. Smell and taste also need development, as many would swallow whatever was placed in the mouth, and would pay no attention to any odour. "And when the hour of instruction is over, the benevolent physician devotes himself to their amusement."

A child admitted at two years of age, a senseless mass, unable to hold up her head, or move her limbs, was so much improved in bodily vigour, in three months, as to be fit for instruction, and a year afterwards was strong, able to walk, and feed herself, knew all parts of the house, could say her letters and many words of one syllable.

A boy, six years old, when admitted could scarcely walk, could not fix his dull eyes on any object, could not speak, and his only sound was like the cry of an animal. It required a month's constant effort before his attention could be directed to any object. A year afterwards his countenance was intelligent, he could walk, feed himself, and pronounce his vowels distinctly. The first three-quarters of a year was given to bodily improvement only.

A girl eight years old, could neither feed herself nor stand eight months ago. Now she has the full use of her limbs, and education is begun. As soon as they are rendered fit to employ themselves in some manual occupation, and to receive ordinary instruction, they are no longer considered as cretins, but are sent to their homes and to the village schools. Three have already left.

Cretinism is often but not always hereditary. "If a child born apparently in good health does not continue to be well developed during the first year, it is observed that nutrition first fails, then the powers of speech and walking, and then the arrest of development becomes complete, if the child is not soon placed in the most advantageous situation." Therefore if the disease is to be arrested, the treatment cannot be begun too early, and one of the rules of the Institution is, that none will be admitted after six years of age, but this is not rigorously adhered to.

We have drawn this sketch from the pamphlet, whose title is prefixed to this notice, and which is written with good sense and good feeling. Dr. W. Twining personally visited the small establishment on the Abendberg, and became so much interested in the undertaking as to endeavour to awake attention to it in England, and a committee is organized to collect subscriptions.

Although we have ourselves no cretins, yet practically this history may be useful by enforcing the great lesson that cannot be learned too often; that hygienic means, pure air, exercise, and diet are the important remedies for restoring muscular strength and nervous energy; that the development of the mental powers is greatly dependent on the due development of the bodily ones, and hence in the weakly or debilitated, the general health must be improved as the necessary preliminary step to education; that inattention in the child to the impressions of the various senses is often a symptom of deficient nervous energy, and one that is to be remedied by improving the bodily strength, and by awakening the attention of each sense, the assiduous and long-continued application of its own appropriate stimulus; and that this "malady of not marking," this deficient power of attention, so injurious to the strength and usefulness of the mind subsequently, should be regarded not indolently as an almost hopeless mental defect, but as a disease which requires for its cure or improvement bodily as well as mental remedies.

It would be an unpardonable neglect to conclude without strongly expressing admiration of Dr. Guggenbuhl. It is a good thing occasionally to find a man, (and one of our own calling,) thus giving up his life to an object of pure, unmixed benevolence; sacrificing everything to a "wish which is the continual subject of his thoughts," when that wish is, not success in life, nor mere honours, nor the carrying out of some scientific object, nor any minor hobby such as men are often possessed by, but to raise to the condition of human beings, a body of his fellow-countrymen who have hitherto been consigned to helpless, hopeless idiotcy.

Dr. Twining's pamphlet is simply, unostentatiously, and agreeably written, bespeaking an acquaintance with his own profession, a cultivated mind, and what is better, a pains-taking interest in the welfare of his species.

